# Exploration of *agr* types, virulence−associated genes, and biofilm formation ability in *Staphylococcus aureus* isolates from hemodialysis patients with vascular access infections

**DOI:** 10.3389/fcimb.2024.1367016

**Published:** 2024-04-12

**Authors:** Chi-Hsiang Lai, Min Yi Wong, Tsung-Yu Huang, Chih-Chen Kao, Yu-Hui Lin, Chu-Hsueh Lu, Yao-Kuang Huang

**Affiliations:** ^1^ Division of Thoracic and Cardiovascular Surgery, Chiayi Chang Gung Memorial Hospital, Chiayi, Taiwan; ^2^ Division of Infectious Diseases, Department of Internal Medicine, Chiayi Chang Gung Memorial Hospital, Chiayi, Taiwan; ^3^ College of Medicine, Chang Gung University, Taoyuan, Taiwan; ^4^ Division of Cardiovascular Surgery, New Taipei Municipal TuCheng Hospital, New Taipei, Taiwan; ^5^ Division of Thoracic and Cardiovascular Surgery, Chiayi Hospital, Ministry of Health and Welfare, Chiayi, Taiwan

**Keywords:** vascular access infections (VAIs), *Staphylococcus aureus*, Agr typing, virulence genes, biofilm formation ability, molecular characterization

## Abstract

**Introduction:**

*Staphylococcus aureus*, is a pathogen commonly encountered in both community and hospital settings. Patients receiving hemodialysis treatment face an elevated risk of vascular access infections (VAIs) particularly *Staphylococcus aureus*, infection. This heightened risk is attributed to the characteristics of *Staphylococcus aureus*, , enabling it to adhere to suitable surfaces and form biofilms, thereby rendering it resistant to external interventions and complicating treatment efforts.

**Methods:**

Therefore this study utilized PCR and microtiter dish biofilm formation assay to determine the difference in the virulence genes and biofilm formation among in our study collected of 103 *Staphylococcus aureus*, isolates from hemodialysis patients utilizing arteriovenous grafts (AVGs), tunneled cuffed catheters (TCCs), and arteriovenous fistulas (AVFs) during November 2013 to December 2021.

**Results:**

Our findings revealed that both MRSA and MSSA isolates exhibited strong biofilm production capabilities. Additionally, we confirmed the presence of agr types and virulence genes through PCR analysis. The majority of the collected isolates were identified as agr type I. However, agr type II isolates displayed a higher average number of virulence genes, with MRSA isolates exhibiting a variety of virulence genes. Notably, combinations of biofilm-associated genes, such as eno−clfA−clfB−fib−icaA−icaD and eno−clfA−clfB−fib−fnbB−icaA−icaD, were prevalent among *Staphylococcus aureus*, isolates obtained from vascular access infections.

**Discussion:**

These insights contribute to a better understanding of the molecular characteristics associated with *Staphylococcus aureus*, infections in hemodialysis patients and provided more targeted and effective treatment approaches.

## 1 Introduction

*Staphylococcus aureus*, a gram-positive bacterium, represents a significant source of infection in both communities and medical institutions. It has developed resistance to a diverse range of antibacterial drugs, giving rise to multi-drug strains such as MRSA (Methicillin-Resistant *Staphylococcus aureus*), presenting considerable challenges in treatment (Lowy, 1998; Taylor and Unakal, 2022). *S. aureus* can colonize various parts of the human body, including the skin, nasal cavity, and more. Upon the onset of wounds, the bacteria invade, leading to skin and soft tissue infections, osteomyelitis, pneumonia, septic arthritis, bacteremia, and endocarditis. Notably, *Staphylococcus aureus* stands as a primary cause of vascular access infection and bacteremia in dialysis patients (Winstel et al., 2019).

Hemodialysis (HD), a life-supporting treatment for individuals with compromised kidney function (Sockrider and Shanawani, 2017), involves three types of vascular access: arteriovenous fistulas (AVFs), arteriovenous grafts (AVGs), and central venous catheters (CVCs) (Maya and Allon, 2008). Despite its life-saving nature, hemodialysis is associated with a heightened risk of morbidity and mortality (LaFrance et al., 2008). During dialysis, patients face an increased risk of intermittent or persistent carriage of *S. aureus*. Moreover, hemodialysis patients with *S. aureus* exhibit a 1.8−4.7 fold higher risk of vascular access infections and bacteremia compared to non-*S. aureus* carriers (Vandecasteele et al., 2009).

Bacterial biofilms, intricate aggregations of bacteria embedded in an extracellular matrix (ECM), pose formidable challenges due to their resistance to mechanical interference, innate and acquired host defenses, and antibiotic treatments. These biofilms contribute significantly to chronic infections, particularly in hospital settings (Costerton et al., 1999). *Staphylococcus aureus*, known for its biofilm-forming capabilities, is notorious for causing chronic infections by resisting therapeutic interventions, especially on indwelling medical devices such as implanted artificial heart valves, catheters, and joint prosthetics (Ribeiro et al., 2012; Moormeier and Bayles, 2017).

Pathogenic *S. aureus* actively engages in the host-pathogen interaction by expressing various virulence genes, which facilitate colonization and infection. These virulence factors, regulated by the accessory gene regulator (agr) locus, categorize *S. aureus* into four groups: type I−IV. These factors not only enable pathogens to enter host tissues, evade immune responses, and attach to host cells but also induce tissue damage through the secretion of exoenzymes and toxins (Kadkhoda et al., 2020; Derakhshan et al., 2021). Microbial surface components recognizing adhesive matrix molecules (MSCRAMMs) on the surface of *S. aureus* play a crucial role in mediating adhesion between bacteria and the host, facilitating essential steps in infection, including clumping factors A, B; fibronectin binding proteins A, B; fibrinogen binding protein; laminin binding protein; collagen binding protein; elastin binding protein; bone sialo-protein binding protein, and ica (intercellular adhesion) operon, mediating cell adhesion and biofilm formation. Additionally, other virulence factors, such as Panton-Valentine leucocidin (PVL), staphylococcal enterotoxins (SEs; SEA to SEE), exfoliative toxins (ETs: ETA and ETB), or toxic shock syndrome toxin-1, are regulated by corresponding genes and contribute to infectious diseases (Mehrotra et al., 2000; Ghasemian et al., 2015; Pakbaz et al., 2017; Idrees et al., 2021; Koosha et al., 2014).

In summary, this study aims to discern the profile of virulence−associated genes, *agr* types, and biofilm formation ability in hemodialysis patients with different dialysis vascular access.

## 2 Methods and materials

### 2.1 Bacterial isolation, collection, and identification

This study was conducted at Chiayi Chang Gung Memorial Hospital in Chiayi, Taiwan. A total of 103 *Staphylococcus aureus* isolates were collected from hemodialysis patients experiencing vascular access infections, including arteriovenous fistulas (AVFs), prosthetic arteriovenous grafts (AVGs), and tunneled–cuffed catheters (TCCs), spanning the period from November 2013 to December 2021. The bacterial isolates were obtained from abscesses, blood, Hickman catheter tips, pus, tissue, and wounds, and cultured on blood agar plates (BAP). Initial identification was accomplished through standard biochemical tests, including catalase and coagulase tests until 2019, with a transition to matrix–assisted laser desorption/ionization time–of–flight mass spectrometry (MALDI–TOF) thereafter. Routine cultivation adhered to laboratory standards on tryptic soy agar and tryptic soy broth, and all isolates were preserved in a 15% glycerol stock at −80°C.

### 2.2 Genomic DNA extraction

A single colony of *S. aureus* was inoculated in Tryptone Soy Broth (TSB) for 16 hours. The overnight culture was centrifuged, and the pellet was resuspended in 1 ml of ultrapure water, heated at 100°C for 15 min, and the supernatant containing DNA was stored at 4°C for subsequent use.

### 2.3 Polymerase chain reaction

PCR amplification, performed in a 25μl reaction mixture, included 1μl of each primer, 2 μl of DNA template, and 12μl of 2x KAPA2G Fast HotStart ReadyMix with dye (Roche, USA). After amplification, the samples were analyzed on a 1.5% agarose gel via electrophoresis, and DNA fragments were visualized using UV light.

### 2.4 Identification of methicillin−resistant *S. aureus*

Isolates were identified as MRSA based on oxacillin resistance and the presence of the *mecA* gene, detected using PCR with previously described primers (Pournajaf et al., 2014). It’s important to note that we classified oxacillin–sensitive *mecA*–positive isolates in this study as MRSA base on a previous study (Hososaka et al., 2007).

### 2.5 Detection of agr types and virulence−associated genes

Amplified *agr* genes were categorized into *agr* types (I−IV) using multiplex PCR (Bibalan et al., 2014). For virulence gene identification, two sets of primers were utilized for multiplex PCR, PCR1 to amplify *bbp*, *cna*, *ebpS*, and *eno* and PCR2 to amplify *fnbA*, *fnbB*, *fib*, *clfA*, and *clfB* (Tristan et al., 2003)., with an additional set designed to amplify *sea*, *seb, sec*, *sed*, *see*, *eta*, *etb*, *tst*, and PVL (Mehrotra et al., 2000; Mcclure et al., 2006). PCR methods for *icaA* and *icaD* genes were also employed (Vasudevan et al., 2003).

### 2.6 Biofilm production ability

The biofilm formation assay followed a previously established protocol (Stepanović et al., 2000). A colony of *S. aureus* was isolated from a tryptone soy agar (TSA) plate and inoculated into tryptone soy broth, then incubated for 12–16 hours at 37°C. The culture was diluted in tryptone soy broth with 0.25% glucose and added to 96 flat–bottom polystyrene wells, followed by incubation for 24 hours at 37°C.

Subsequently, the planktonic cells were aspirated, and the plate was washed twice with sterile saline water to remove non–adherent bacterial cells. The attached bacteria were fixed with 99% methanol for 15 minutes, then the plates were emptied and air–dried. Next, 0.1% crystal violet was added to each well, and after 15 minutes, the excess crystal violet was removed by washing the plate twice with water and air–drying.

Finally, the cell–bound crystal violet was dissolved in 99% ethanol and allowed to stand for 15 minutes. Optical density (OD) of the isolates was monitored by measuring absorbance at 570 nm using a microplate reader (Perkin Elmer Enspire 2300, Perkin Elmer, USA). The negative control consisted of only broth, while the biofilm‐forming S. aureus reference strain, ATCC 29213, was used as the positive control.

The adherence capabilities of the tested isolates were classified into four categories following the method described by previous research (Christensen et al., 1985). The cut–off value (ODc) was established as three standard deviations (SD) above the mean OD of the negative control: ODc = average OD of negative control + (3 × SD of negative control).

Strains were classified into the following categories:

OD ≤ ODc = no biofilm producer,
ODc < OD ≤ 2 × ODc = weak biofilm producer,
2 × ODc < OD ≤ 4 × ODc = moderate biofilm producer,
4 × ODc < OD = strong biofilm producer.

## 3 Results

### 3.1 The ratio of methicillin−resistant *S. aureus* and methicillin−sensitive *S. aureus* from different vascular access infections

**Figure 1:**
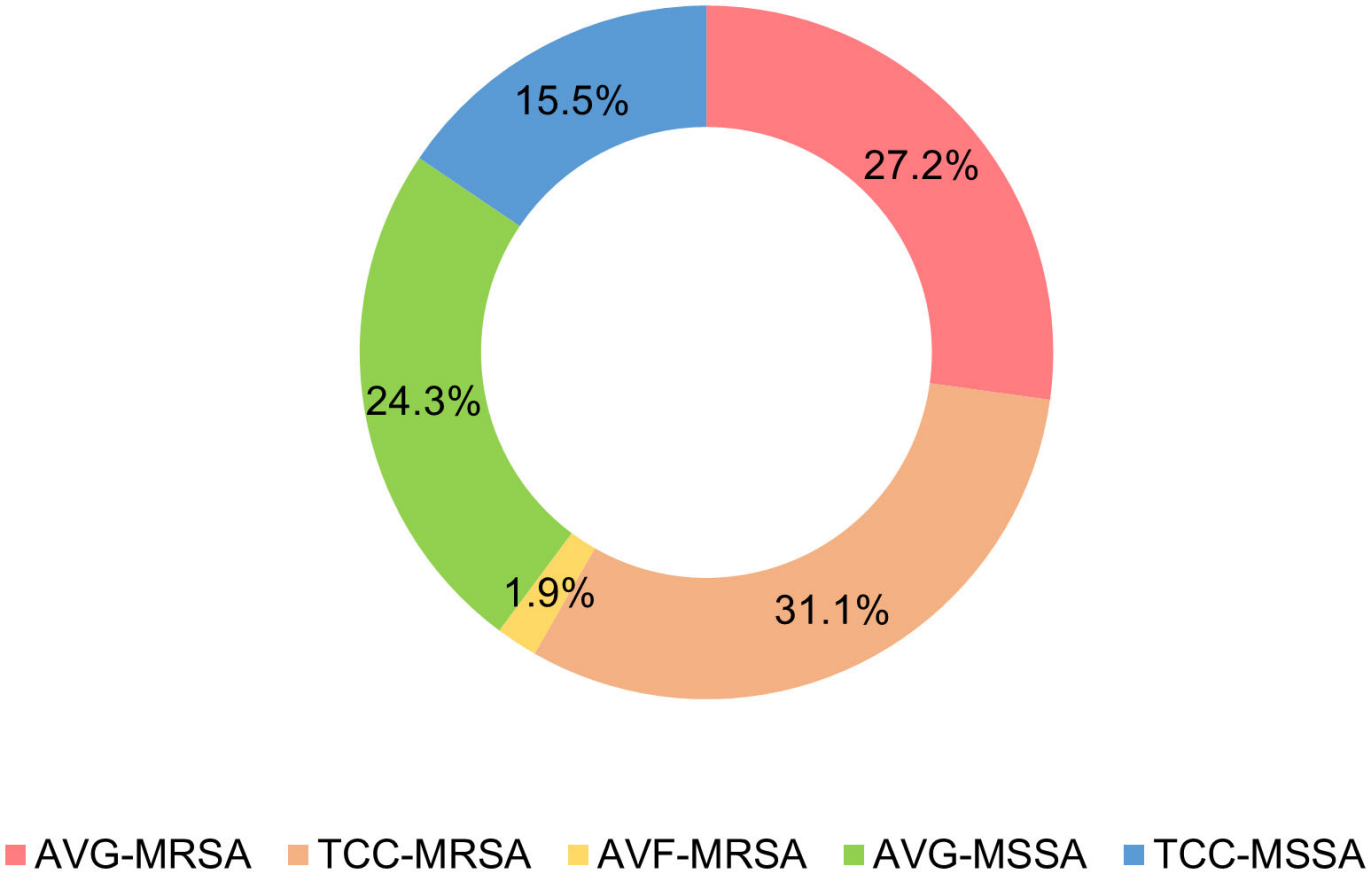
Distribution of *Staphylococcus aureus* isolates from vascular access infections. AVF, arteriovenous fistulas, AVG, prosthetic arteriovenous grafts, TCC, tunneled–cuffed catheters, MRSA, methicillin–resistant *S. aureus*, MSSA, methicillin–sensitive *S. aureus*.

A total of 103 *Staphylococcus aureus* isolates were collected from three types of vascular access infections (**Figure 1**). The majority of isolates were TCC–MRSA and AVG–MRSA, constituting 31.1% (32/103) and 27.2% (28/103), respectively. Following were 24.3% (25/103) AVG–MSSA and 15.5% (16/103) TCC–MSSA, with AVF–MRSA being less prevalent at only 1.9% (2/103). Some of the isolates, collected from different sites among the 103 *S. aureus* isolates, belong to the same patients, therefore, they were be considered as individual isolates. Furthermore, the age range of patients was predominantly between 40 and 90 years old. Females more than males, and AVG−*S. aureus* isolates were predominantly from female patients. Regarding specimens, the majority of AVF and AVG−*S. aureus* isolates were found in blood, pus, and wound samples, whereas TCC−*S. aureus* isolates were primarily from tip. This disparity could be associated with the pattern of dialysis access (**Table 1**).

**Table 1:** The characteristics of S. aureus isolates from hemodialysis patients.

		AVF–MRSA (2) (%)	AVG–MRSA (28) (%)	AVG–MSSA (25) (%)	TCC–MRSA (32) (%)	TCC–MSSA (16) (%)
Age	0~10					
	11~20					
	21~30					
	31~40				1	
	41~50			1	3	2
	51~60		6	4	4	2
	61~70	1	5	10	7	8
	71~80		6	3	5	1
	81~90		2	2	2	2
	91~100				2	
Sex	male	0	5	5	12	9
	female	1	14	15	12	6
Isolation	blood	1 (50)	4 (14.3)	5 (20)	7 (21.9)	0 (0)
	abscess	0 (0)	3 (10.7)	0 (0)	0 (0)	0 (0)
	pus	0 (0)	9 (32.1)	6 (24)	3 (9.4)	2 (12.5)
	tip	0 (0)	1 (3.6)	2 (8)	20 (62.5)	14 (87.5)
	tissue	0 (0)	2 (7.1)	4 (16)	0 (0)	0 (0)
	wound	1 (50)	9 (32.1)	8 (32)	2 (6.3)	0 (0)

### 3.2 Distribution of agr types of S. aureus isolates from vascular access infections

**Figure 2:**
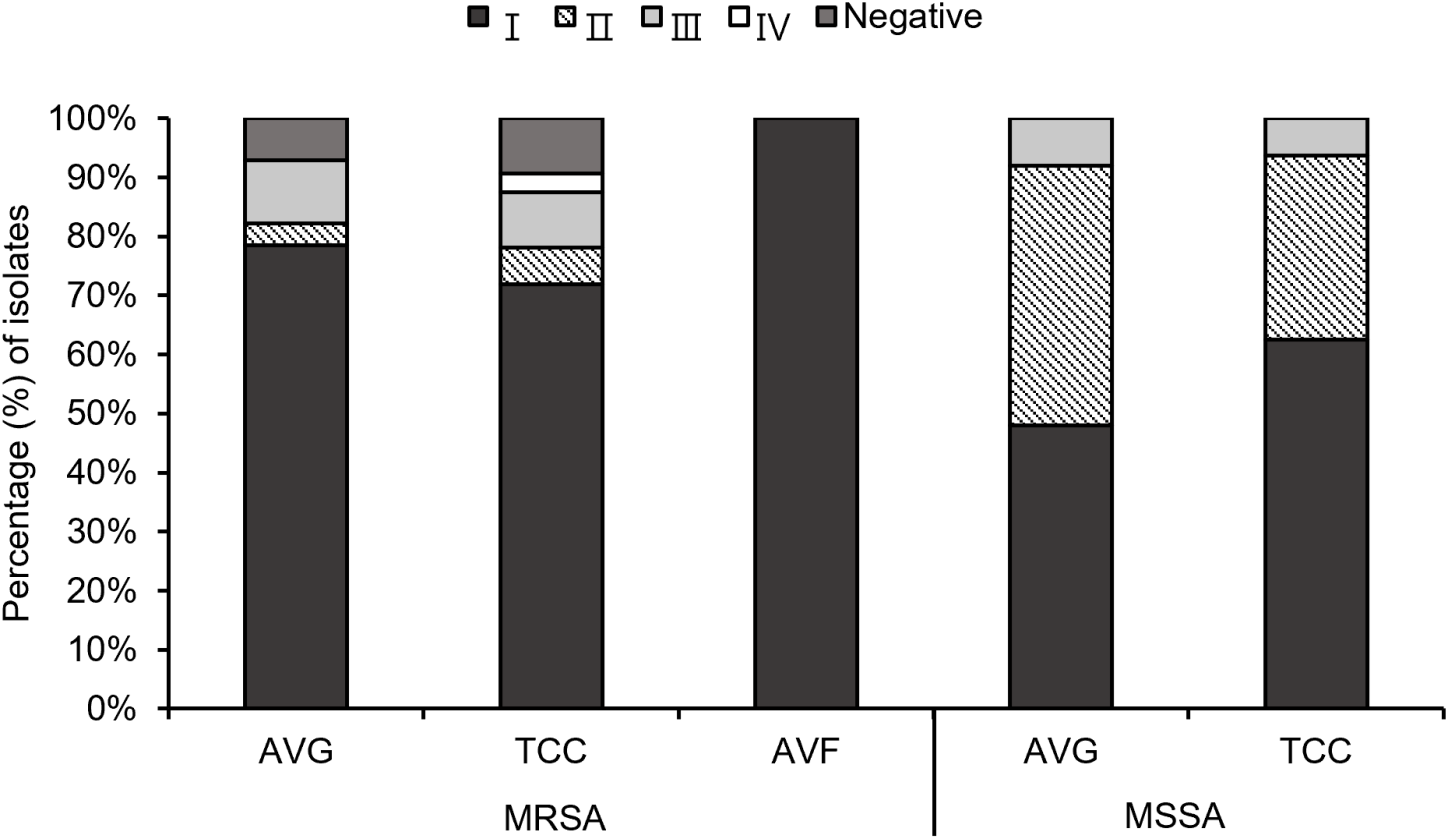
The profile of *agr* types among methicillin–resistant *S. aureus* (MRSA) and methicillin–sensitive *S. aureus* (MSSA) isolates from different vascular access infections.

Through multiplex PCR, four *agr* types were investigated among the 103 *S. aureus* isolates (**Figure 2**). A small number of MRSA isolates, specifically 7.1% (2/28) AVG–MRSA and 9.4% (3/32) TCC–MRSA, were non–typeable for the *agr* locus, labeled as *agr–*negative isolates. *Agr* I was prevalent in AVG–MRSA, TCC–MRSA, AVF–MRSA, AVG–MSSA, and TCC–MSSA, constituting 78.6% (22/28), 71.9% (23/32), 100% (2/2), 48% (12/25), and 62.5% (10/16), respectively. *Agr* II was the secondary prevalent type in MSSA isolates, with AVG–MSSA harboring 44.0% (11/25) and TCC–MSSA harboring 31.3% (5/16). *Agr* III was the following prevalent type in MRSA isolates, constituting 10.7% (3/28) AVG–MRSA and 9.4% (3/32) TCC–MRSA. *Agr* IV was only detected in TCC–MRSA at 3.1% (1/32).

### 3.3 The prevalence of virulence−associated genes among *S. aureus* isolates from different vascular access infections

**Figure 3:**
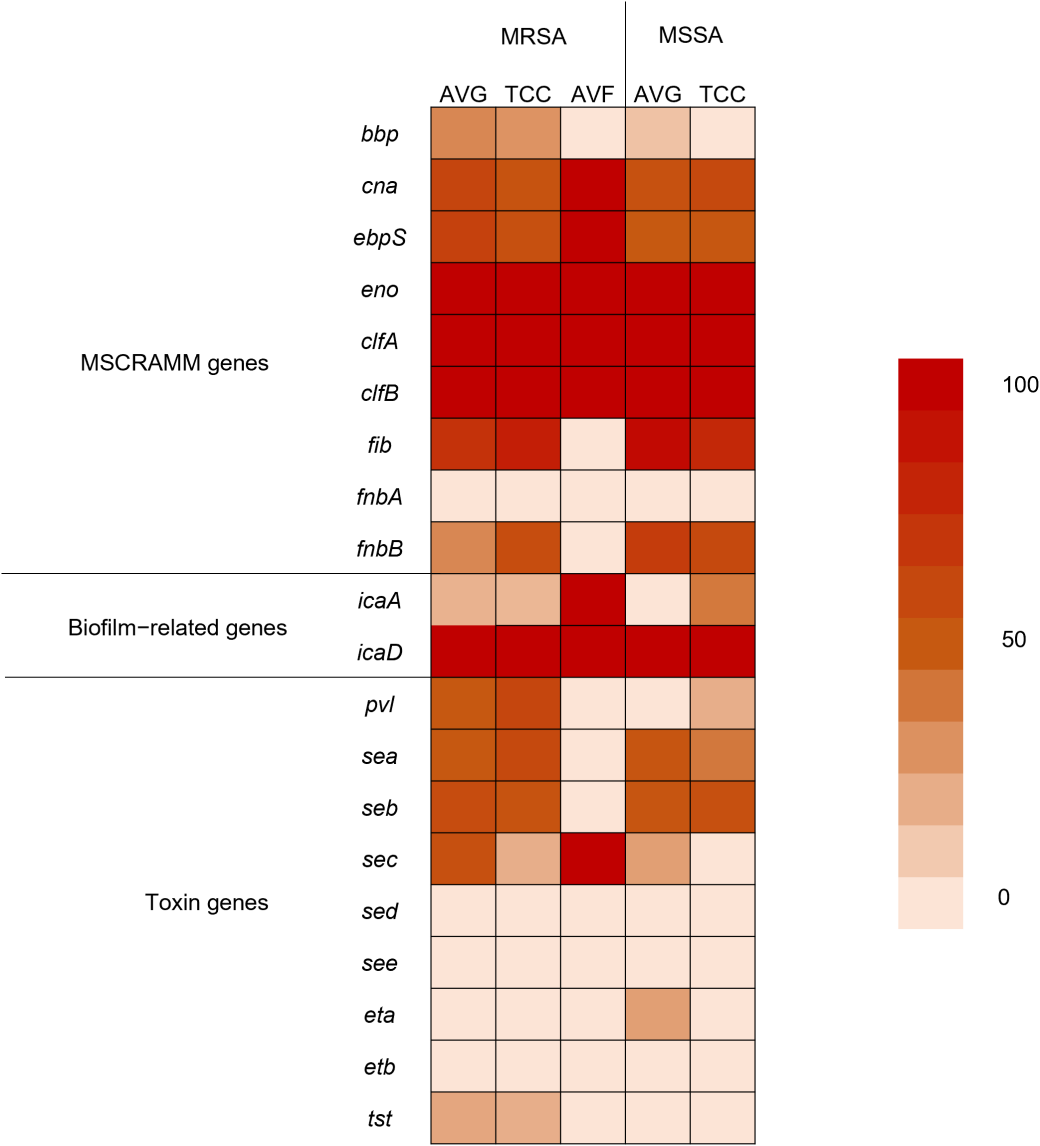
Heatmap indicating the prevalence of virulence genes among methicillin–resistant *S. aureus* (MRSA) and methicillin–sensitive *S. aureus* (MSSA) isolates from different vascular access infections.

Using PCR to detect 20 virulence−associated genes, *eno* (100%), *clfA* (100%), *clfB* (100%), and *icaD* (100%) genes were prevalent in all *S. aureus* isolates from different vascular access infections. Following were *fib* (68.9%), and *icaA* (81.5%) genes. *fnbA*, *sed*, *see*, and *etb* genes were not observed in *S. aureus* isolates (**Figure 3**). The variety of virulence genes was more pronounced among AVG–MRSA and TCC–MRSA than MSSA isolates. Furthermore, it is evident that *eno*, *clfA*, and *clfB* genes were prevalent in *S. aureus* isolates from blood and other sites, followed by *fib* genes. However, except for the *bbp* genes, which were only detected in *S. aureus* isolates from other sites, the quantity of virulence genes in *S. aureus* isolates showed minimal differences between blood sites and other sites (**Table 2**).

**Table 2:** The profile of virulence genes in *S. aureus* isolates from blood and others specimen site.

	MRSA	MSSA	All	MRSA	MSSA	All
	Blood (12) (%)	Blood (5) (%)	Blood (17) (%)	Others (50) (%)	Others (36) (%)	Others (86) (%)
*bbp*	0 (0)	0 (0)	0 (0)	6 (12)	1 (2.8)	7 (8.1)
*cna*	7 (58.3)	0 (0)	7 (41.2)	12 (24)	11 (30.6)	23 (26.7)
*ebpS*	6 (50)	0 (0)	6 (35.3)	15 (30)	7 (19.4)	22 (25.6)
*eno*	12 (100)	5 (100)	17 (100)	50 (100)	36 (100)	86 (100)
*clfA*	12 (100)	5 (100)	17 (100)	50 (100)	36 (100)	86 (100)
*clfB*	12 (100)	5 (100)	17 (100)	50 (100)	36 (100)	86 (100)
*fib*	7 (58.3)	5 (100)	12 (70.6)	31 (62)	28 (77.8)	59 (68.6)
*fnbA*	0 (0)	0 (0)	0 (0)	0 (0)	0 (0)	0 (0)
*fnbB*	2 (16.7)	3 (60)	5 (29.4)	10 (20)	13 (36.1)	23 (26.7)
*pvl*	2 (16.7)	0 (0)	2 (11.8)	14 (28)	1 (2.8)	15 (17.4)
*sea*	2 (16.7)	0 (0)	2 (11.8)	13 (26)	7 (19.4)	20 (23.3)
*seb*	2 (16.7)	1 (20)	3 (17.6)	13 (26)	8 (22.2)	21 (24.4)
*sec*	4 (33.3)	0 (0)	4 (23.5)	7 (14)	2 (5.6)	9 (10.5)
*sed*	0 (0)	0 (0)	0 (0)	0 (0)	0 (0)	0 (0)
*see*	0 (0)	0 (0)	0 (0)	0 (0)	0 (0)	0 (0)
*eta*	0 (0)	1 (20)	1 (5.9)	0 (0)	0 (0)	0 (0)
*etb*	0 (0)	0 (0)	0 (0)	0 (0)	0 (0)	0 (0)
*tst*	1 (8.3)	0 (0)	1 (5.9)	3 (6)	0 (0)	3 (3.5)
*icaA*	10 (83.3)	4 (80)	14 (82.4)	41 (82)	29 (80.6)	70 (81.4)
*icaD*	12 (100)	5 (100)	17 (100)	50 (100)	36 (100)	86 (100)

### 3.4 Distribution of the average number of virulence−associated genes among *S. aureus* isolates from vascular access infections in different *agr* types

**Figure 4:**
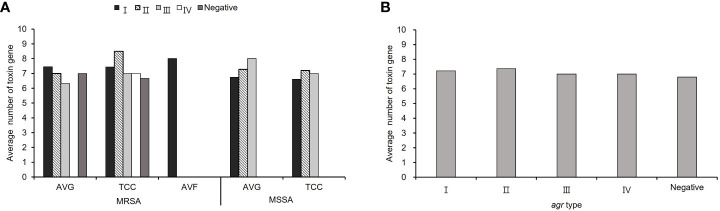
The virulence−associated gene content of methicillin–resistant *S. aureus* (MRSA) and methicillin–sensitive *S. aureus* (MSSA) isolates in different *agr* types. **(A)** The average number of virulence genes from *S. aureus* with different vascular access infections. **(B)** The average number of virulence genes in the *agr*− positive and negative isolates among all *S. aureus* isolates.

The average number of virulence−associated genes was investigated according to *agr* genotype, calculated as the total number of genes in each *agr* type with different vascular access infections divided by the total number of isolates in each *agr* type with different vascular access infections. The average number of virulence genes was 7.23 in all *S. aureus* isolates. Among MRSA isolates, *agr* I and II was harbored most average number of virulence gene (I: 7.45 for AVG−MRSA, 8 for AVF−MRSA, II: 8.5 for TCC−MRSA), in MSSA isolates, *agr* II and III had most average number of virulence gene (II: 7.2 for TCC−MSSA and III: 8 for AVG−MSSA). A lower average number of virulence genes was detected in *agr*−negative isolates compared to *agr*−positive isolates among TCC–MRSA (**Figure 4A**). In all *S. aureus* isolates, the content of virulence−associated genes, *agr* II isolates had the highest average number of virulence genes (averaging 7.37), whereas *agr*−negative isolates had the lowest average number of virulence genes (averaging 6.8) (**Figure 4B**).

### 3.5 Biofilm production ability of *S. aureus* isolates from vascular access infections

**Figure 5:**
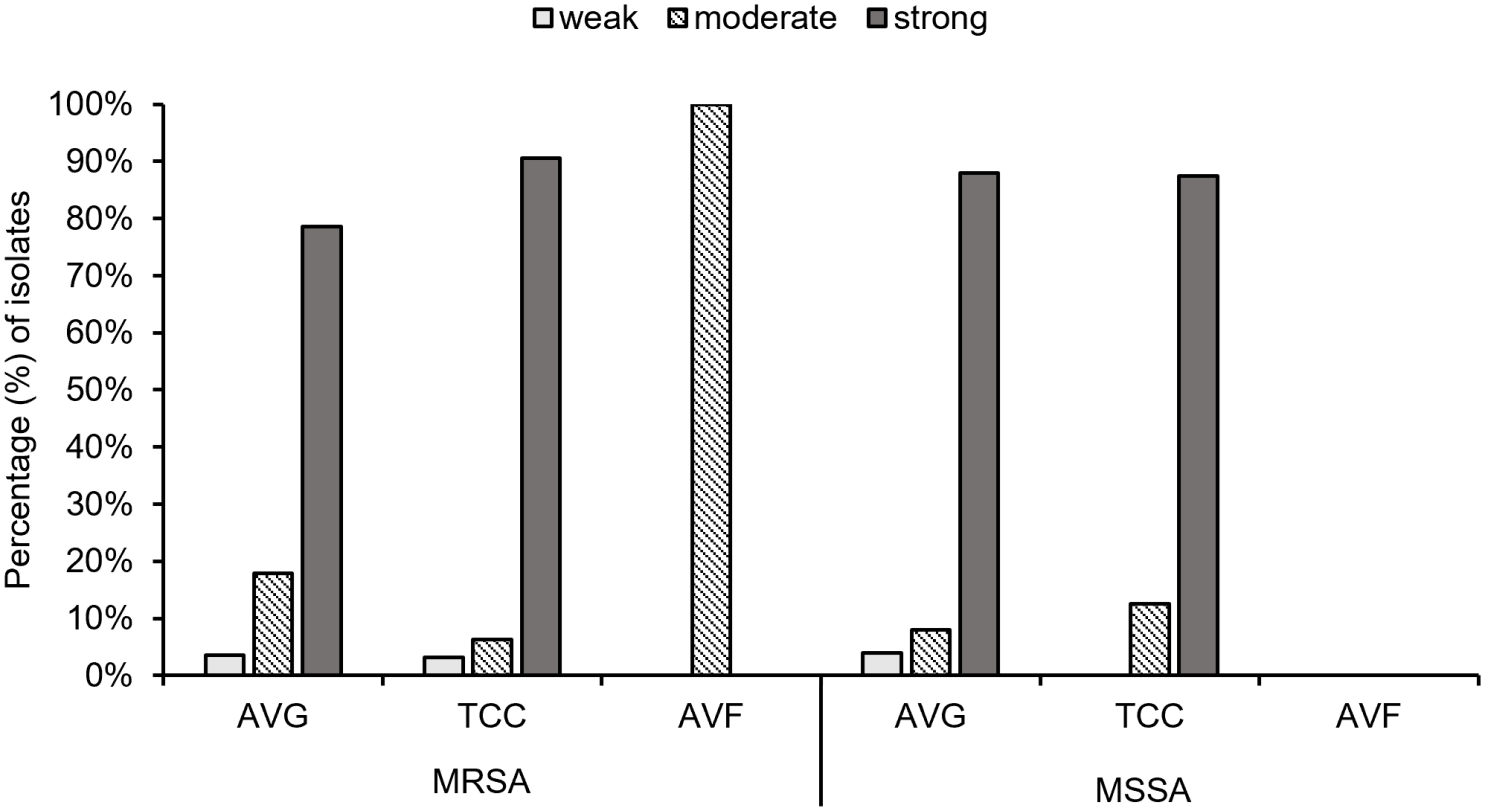
The biofilm production ability of methicillin–resistant *S. aureus* (MRSA) and methicillin–sensitive *S. aureus* (MSSA) isolates from different vascular access infections.

Biofilm production ability was analyzed, and all *S. aureus* isolates from vascular access infections were able to produce biofilm (**Figure 5**). Over 75% of MRSA and MSSA isolates exhibited a strong biofilm production ability after 24 hours of incubation, including 78.6% (22/28) AVG–MRSA, 90.6% (29/32) TCC–MRSA, 88.0% (22/25) AVG–MSSA, and 87.5% (14/16) TCC–MSSA. All AVF–MRSA isolates produced a moderate biofilm ability. The results indicated no significant difference in biofilm production ability between MRSA and MSSA, with most MRSA and MSSA demonstrating strong biofilm–forming capabilities.

### 3.6 The correlation of *agr* types and biofilm production ability

Both *agr*–positive and *agr*–negative isolates exhibited strong biofilm formation ability. *Agr* type II isolates collected were all strong biofilm producers, while *agr* type III isolates collected, except AVG–MRSA, were all strong biofilm producers. *Agr* type I isolates with strong biofilm production ability also demonstrated moderate and weak biofilm production ability among all *S. aureus* isolates (**Table 3**).

**Table 3:** The relationship between different types of *agr* and biofilm formation ability among methicillin–resistant *S. aureus* (MRSA) and methicillin–sensitive *S. aureus* (MSSA) isolates from vascular access infections.

			Biofilm production ability			
		agr types	Weak (%)	Moderate (%)	Strong (%)	NO.
MRSA	AVG (n=28)	I	1 (4.5)	4 (18.2)	17 (77.3)	22
		II	0 (0)	0 (0)	1 (100)	1
		III	0 (0)	1 (33.3)	2 (66.7)	3
		IV	0 (0)	0 (0)	0 (0)	0
		negative	0 (0)	0 (0)	2 (100)	2
	TCC (n=32)	I	0 (0)	1 (4.3)	22 (95.7)	23
		II	0 (0)	0 (0)	2 (100)	2
		III	0 (0)	0 (0)	3 (100)	3
		IV	0 (0)	1 (100)	0 (0)	1
		negative	1 (33.3)	0 (0)	2 (66.7)	3
	AVF (n=2)	I	0 (0)	2 (100)	0 (0)	2
		II	0 (0)	0 (0)	0 (0)	0
		III	0 (0)	0 (0)	0 (0)	0
		IV	0 (0)	0 (0)	0 (0)	0
		negative	0 (0)	0 (0)	0 (0)	0
MSSA	AVG (n=25)	I	1 (8.3)	2 (16.7)	9 (75)	12
		II	0 (0)	0 (0)	11 (100)	11
		III	0 (0)	0 (0)	2 (100)	2
		IV	0 (0)	0 (0)	0 (0)	0
		negative	0 (0)	0 (0)	0 (0)	0
	TCC (n=16)	I	0 (0)	2 (20)	8 (80)	10
		II	0 (0)	0 (0)	5 (100)	5
		III	0 (0)	0 (0)	1 (100)	1
		IV	0 (0)	0 (0)	0 (0)	0
		negative	0 (0)	0 (0)	0 (0)	0

### 3.7 The correlation between biofilm–related gene combinations and biofilm production ability among *S. aureus* isolates

A comprehensive analysis was conducted to examine the relationship between 18 combinations of biofilm−related genes and their association with biofilm production capacity among *S. aureus* isolates (**Table 4**). The most prevalent gene patterns contributing to strong biofilm production were *eno*−*clfA*−*clfB*−*fib*−*icaA*−*icaD* (18.4%, 19/103) and *eno*−*clfA*−*clfB*−*fib*−*fnbB*−*icaA*−*icaD* (18.4%, 19/103). These patterns were observed in isolates encompassing AVG–MRSA, TCC–MRSA, AVG–MSSA, and TCC–MSSA. Secondary patterns of virulence genes, such as *cna*−*ebpS*−*eno*−*clfA*−*clfB*−*icaA*−*icaD* (11.6%, 12/103), were primarily found in MRSA isolates, correlating with both moderate and weak biofilm production capabilities.

**Table 4:** The relationship between biofilm−related gene combinations and biofilm production capacity of methicillin–resistant *S. aureus* (MRSA) and methicillin–sensitive *S. aureus* (MSSA) isolates in vascular access infections.

	MRSA							MSSA					total
	weak		moderate			strong		weak	moderate		strong		
	AVG	TCC	AVG	TCC	AVF	AVG	TCC	AVG	AVG	TCC	AVG	TCC	
*cna*, *ebpS*, *eno*, *clfA*, *clfB*, *icaA*, *icaD*	1	1	4	1	2	2	1	0	0	0	0	0	12
*cna*, *eno*, *clfA*, *clfB*, *fib*, *icaA*, *icaD*	0	0	0	0	0	2	1	0	0	0	3	3	9
*cna*, *eno*, *clfA*, *clfB*, *fib*, *fnbB*, *icaA*, *icaD*	0	0	0	0	0	1	3	0	0	0	0	0	4
*cna*, *eno*, *clfA*, *clfB*, *icaD*	0	0	0	0	0	0	0	0	0	0	1	0	1
*cna*, *eno*, *clfA*, *clfB*, *icaA*, *icaD*	0	0	0	0	0	0	0	0	0	0	0	1	1
*cna*, *eno*, *clfA*, *clfB*, *fib*, *icaD*	0	0	0	0	0	0	0	0	0	0	0	1	1
*cna*, *ebpS*, *eno*, *clfA*, *clfB*, *fib*, *icaA*, *icaD*	0	0	0	0	0	0	0	0	0	0	2	0	2
*ebpS*, *eno*, *clfA*, *clfB*, *icaD*	0	0	1	0	0	1	1	0	0	0	0	0	3
*ebpS*, *eno*, *clfA*, *clfB*, *icaA*, *icaD*	0	0	0	0	0	1	2	0	0	0	1	2	6
*ebpS*, *eno*, *clfA*, *clfB*, *fib*, *icaA*, *icaD*	0	0	0	0	0	1	1	0	0	0	0	0	2
*ebpS*, *eno*, *clfA*, *clfB*, *fib*, *icaD*	0	0	0	0	0	0	1	0	0	0	0	0	1
*ebpS*, *eno*, *clfA*, *clfB*, *fib*, *fnbB*, *icaD*	0	0	0	0	0	0	0	1	0	0	0	0	1
*ebpS*, *eno*, *clfA*, *clfB*, *fib*, *fnbB*, *icaA*, *icaD*	0	0	0	0	0	0	0	0	0	1	0	0	1
*eno*, *clfA*, *clfB*, *icaA*, *icaD*	0	0	0	0	0	3	3	0	0	0	0	3	9
*eno*, *clfA*, *clfB*, *fib*, *icaD*	0	0	0	0	0	3	4	0	1	0	1	0	9
*eno*, *clfA*, *clfB*, *fib*, *icaA*, *icaD*	0	0	0	1	0	6	6	0	1	1	4	0	19
*eno*, *clfA*, *clfB*, *fib*, *fnbB*, *icaA*, *icaD*	0	0	0	0	0	2	6	0	0	0	7	4	19
*eno*, *clfA*, *clfB*, *fib*, *fnbB*, *icaD*	0	0	0	0	0	0	0	0	0	0	3	0	3

## 4 Discussion

*Staphylococcus aureus* carriage is a pivotal factor in *Staphylococcus aureus* infections among dialysis patients, contributing to increased morbidity and mortality in both hemodialysis and peritoneal dialysis settings. Carriage of *Staphylococcus aureus* in the anterior nasal cavity emerges as a significant risk factor for infections. Prior investigations have revealed that 40%−60% of hemodialysis patients harbor *Staphylococcus aureus* in the anterior nasal cavity, with a carriage rate and infection risk double that of healthy controls. Molecular typing of strains carried by individuals has shown that strains from the nasal cavity, skin, and infection sites match, indicating bacterial transmission from the nose to hands and subsequently to the skin, causing infections through foreign bodies such as grafts (introduced through venipuncture) or dialysis catheters (HD or PD). The entry of catheters may result in contamination during catheter insertion or through the tunnel at the exit site, leading to access site infections (Kirmani, 1978; Piraino, 2000; Balaban et al., 2003). In this study, we collected 145 *Staphylococcus aureus* isolates, of which 71% (103/145) were sourced from vascular access infections. The ratio of infections from arteriovenous grafts (AVG) to tunneled–cuffed catheters (TCC) infections was approximately 1:1, with sporadic isolates from arteriovenous fistula (AVF) infections.

Biofilm formation is a critical adaptive and survival strategy employed by bacteria, occurring on both biotic and abiotic surfaces in various environments, including healthcare settings. Extracellular polymeric substances (EPS) produced during biofilm formation protect bacteria from adverse environmental factors and immune responses (Kostakioti et al., 2013; Tan et al., 2014). Bacteria enclosed in biofilms can adhere to wounds, hindering the healing process of chronic wounds and producing toxins that impede wound healing or confer resistance to antibiotics (Rajpaul, 2015). Previous studies have implicated biofilm formation as a risk factor for bacterial infections in hemodialysis patients, with *Staphylococcus aureus* identified as a great biofilm producer (Marques et al., 2017; Kwiecinski et al., 2019). Our study confirms that all *Staphylococcus aureus* isolates from vascular access infections demonstrated biofilm formation ability, with 84.5% (87/103) classified as strong biofilm producers. Among these, MRSA isolates (58.6%, 51/87) exhibited a higher prevalence of strong biofilm production compared to MSSA isolates (41.4%, 36/87), particularly TCC–MRSA, with 33.3% (29/87) of strong biofilm isolates.

Biofilm formation involves microbial cell attachment to surfaces and subsequent accumulation, a key factor in infection development. Adhesion formation, a major virulence factor of *Staphylococcus aureus*, is correlated with various genes, including *bbp*, *cna*, *ebpS*, *eno*, *clfA*, *clfB*, *fib*, *fnbA*, *fnbB*, and the ica group genes. Previous studies have identified *clfA*, *clfB*, *fib*, *eno*, *icaA*, and *icaD* genes as prevalent in *Staphylococcus aureus* isolates (Atshan et al., 2012; Ghasemian et al., 2015; Xu et al., 2021). Our study yielded similar results, with all MRSA and MSSA isolates harboring *eno*, *clfA*, *clfB*, and *icaD* genes, followed by *fib* and *icaA* genes. However, *fnbA* genes were not detected in any isolates, diverging from previous research. *Staphylococcus aureus* infections are also associated with various virulence genes, including Panton–Valentine leukocidin (PVL), toxic shock syndrome toxin–1 (TSST–1), exfoliative toxins (ETs), and staphylococcal enterotoxins (SEs). Although PVL is linked to leukocyte cytolysis, and SEs and TSST–1 primarily cause food poisoning and toxic shock syndrome, our study identified a low frequency of *pvl*, *tst*, *eta*, *etb*, *sea*, *seb*, *sec*, *sed*, and *see* genes in all *Staphylococcus aureus* isolates, with *eta*, *etb*, *tst*, *sed*, and *see* genes nearly absent, aligning with previous reports (Wang et al., 2021).

Patients infected with *Staphylococcus aureus* may experience various illnesses, including sepsis, pneumonia, septic arthritis, osteomyelitis, toxic shock syndrome following surgery, folliculitis, endocarditis, and urinary tract infections. The accessory gene regulator (agr) system plays a crucial role in controlling and regulating the expression of virulence genes in *Staphylococcus aureus*. This system classifies *S. aureus* isolates into four groups (type I, II, III, IV) based on amino acid polymorphisms of AgrB, AgrD, and AgrC. Several studies have associated specific diseases with different *agr* groups, such as invasive infections linked to *agr* group I strains and non−invasive infections to *agr* group III strains. Notably, *agr* group I predominates among *S. aureus* isolates (Bibalan et al., 2014; Javdan et al., 2019; Tan et al., 2022). Our study observed a prevalence of *agr* type I (67%, 69/103) in *S. aureus* isolates from vascular access infections, particularly in MRSA isolates. Calculations of the average number of virulence genes in each *agr* type revealed that *agr* II isolates had the highest average number of virulence genes, consistent with previous studies (Zhang et al., 2018). Additionally, MRSA isolates exhibited a slightly higher average number of virulence genes (8.1, 502/62) than MSSA isolates (7.9, 324/41).

Correlations between *agr* group and biofilm formation, as well as between virulence genes and biofilm formation, were explored. Previous studies have identified *agr* groups II and III as the primary biofilm producers among the four types, with *agr* type II in MRSA exhibiting superior biofilm formation ability (Tan et al., 2018). Our study corroborates these findings, revealing that *agr* group II constituted strong biofilm producers among collected *S. aureus* isolates from various vascular infections. The prevalence of biofilm−associated genes, specifically the combination of *fib*−*eno*−*clfA*−*clfB*−*ebpS*−*icaA*−*icaD* (Kaźmierczak et al., 2021), aligns with previous reports, with *eno*−*clfA*−*clfB*−*fib*−*icaA*−*icaD* and *eno*−*clfA*−*clfB*−*fib*−*fnbB*−*icaA*−*icaD* being prevalent gene patterns in our study. The following pattern, *cna*−*ebpS*−*eno*−*clfA*−*clfB*−*icaA*−*icaD*, was also observed. Thus, *eno*, *clfA*, *clfB*, *icaA*, and *icaD* genes appear more frequently in biofilm–producing isolates of *Staphylococcus aureus*.

While this study investigated the molecular, phenotypic, and genotypic characteristics of *S. aureus* isolates from different vascular access infections, it has limitations. Conducted over an 8–year period at a single institution, the varying frequencies of isolates collected each year and the small sample size may introduce bias.

## 5 Conclusion

All the MRSA and MSSA isolates obtained from vascular access infections (VAIs) in our study exhibited biofilm–forming capabilities, particularly demonstrating strong biofilm capacity. Subsequent PCR identification confirmed that the majority of *Staphylococcus aureus* isolates belonged to *agr* type I. Additionally, the analysis of virulence genes revealed that AVG−MRSA and TCC−MRSA isolates displayed the most diverse array of virulence genes. Molecular testing of *Staphylococcus aureus* in the context of vascular access infections is crucial for advancing infection management and developing effective treatment strategies.

## Data availability statement

All experimental data during this study are included in this published article.

## Ethics statement

The studies were approved by Institutional Review Board (IRB) of Chang Gung Memorial Hospital (IRB201508482B0 and IRB201901354B0). The studies were conducted in accordance with the local legislation and institutional requirements. The bacteria samples used in this study were acquired from Department of Laboratory Medicine from the Chang Gung Memorial Hospital, Chiayi, Taiwan. Written informed consent for participation was obtained from the participants or the participants’ legal guardians/next of kin in accordance with the national legislation and institutional requirements.

## Author contributions

Y–KH: Conceptualization, Funding acquisition, Methodology, Project administration, Resources, Supervision, Writing – original draft, Writing – review & editing. C–HLa: Data curation, Formal analysis, Investigation, Writing – original draft, Writing – review & editing. MW: Data curation, Formal analysis, Investigation, Writing – original draft, Writing – review & editing. T–YH: Funding acquisition, Methodology, Resources, Writing – review & editing. C–CK: Conceptualization, Funding acquisition, Project administration, Writing – review & editing. Y–HL: Investigation, Validation, Writing – review & editing. C–HLu: Investigation, Validation, Writing – review & editing.
